# Safety Profile of Intraoperative Corneal Debridement in Descemet Membrane Endothelial Keratoplasty (DMEK)—A Retrospective Comparative Study

**DOI:** 10.1155/joph/6694690

**Published:** 2025-12-19

**Authors:** Sebastian Arens, Stefan J. Lang, Daniel Böhringer, Thomas Reinhard

**Affiliations:** ^1^ Eye Center, Medical Center, Faculty of Medicine, University of Freiburg, Freiburg im Breisgau, Germany, uni-freiburg.de; ^2^ Department of Ophthalmology, Brandenburg Medical School, University Hospital Brandenburg, Brandenburg an der Havel, Germany

**Keywords:** corneal debridement, DMEK, endothelial cell density, Fuchs’ endothelial dystrophy, graft survival, visual outcomes

## Abstract

**Purpose:**

To investigate the influence of intraoperative corneal debridement on postoperative outcomes in Descemet Membrane Endothelial Keratoplasty (DMEK).

**Methods:**

This retrospective comparative study analyzed 3168 eyes that underwent DMEK between 2012 and 2024. Intraoperative corneal debridement was performed in 215 eyes (6.8%) to improve visualization during the procedure. Survival time analyses with Kaplan–Meier curves and log‐rank test and multifactorial Cox regression were conducted to assess the impact of corneal debridement on postoperative graft survival.

**Results:**

Bivariate survival analyses suggested that intraoperative corneal debridement during DMEK may be associated with worse postoperative outcomes in terms of visual acuity, endothelial cell density, graft survival, and perioperative complications. Using the log‐rank test, significant differences were observed in endothelial cell density, postoperative regrafting, and visual acuity (*p* < 0.005), while the need for rebubbling was not statistically significant (*p* = 0.5). However, multifactorial Cox regression analysis, controlling for potential confounding factors, revealed that the difference in hazard ratios between the debridement and no‐debridement groups had no statistically significant effect on graft survival (HR = 0.74, 95% CI: 0.49–1.11, *p* = 0.141). Patients with Fuchs’ endothelial dystrophy had a significantly lower risk of graft failure compared to other indications (HR 0.41, *p* < 0.001).

**Conclusions:**

In bivariate analyses, intraoperative corneal debridement was associated with worse postoperative outcomes, including reduced visual acuity, endothelial cell density, and graft survival. However, these differences likely reflect the more severe cases in the debridement group. When adjusting for potential confounders, multifactorial Cox regression analysis showed that corneal debridement did not have a significant impact on graft survival. This suggests that while debridement may be associated with poorer outcomes in unadjusted analyses, it does not appear to adversely affect graft survival when accounting for other factors. Corneal debridement may therefore be a safe and viable option in DMEK, though further research is needed to confirm its impact on long‐term outcomes.

## 1. Introduction

Descemet Membrane Endothelial Keratoplasty (DMEK) has emerged as the preferred surgical technique for treating corneal endothelial disorders, such as Fuchs’ endothelial dystrophy and pseudophakic bullous keratopathy. The corneal endothelium, a single layer of cells lining the inner corneal surface, is crucial for maintaining corneal transparency by regulating hydration. Endothelial compromise can lead to corneal edema, decreased visual acuity, and reduced quality of life. DMEK selectively replaces the diseased endothelium and Descemet membrane with a healthy donor graft, offering faster visual recovery and better outcomes than previous techniques such as Descemet Stripping Automated Endothelial Keratoplasty (DSAEK) [1–4].

However, DMEK is technically challenging, requiring precise manipulation of the delicate donor tissue within the anterior chamber. In cases with advanced corneal edema or poor visualization, surgeons may perform intraoperative corneal debridement of the corneal epithelium to improve visualization and facilitate graft positioning. This technique is especially necessary when poor visual conditions (due to bullous keratopathy or extensive corneal edema) would otherwise risk safe surgery. Corneal debridement involves mechanically removing the corneal epithelium to temporarily enhance surgical field clarity. Despite its potential benefits mainly because of better intraoperative vision, concerns may exist about corneal debridement’s impact on postoperative outcomes, including visual acuity recovery, endothelial cell loss, and graft survival as well as hospitalization time. This is especially due to the fact that corneal debridement might lead to an easier invasion of bacteria or to a prolonged inflammation of corneal tissue that might show an effect on the aforementioned outcomes. Corneal debridement is a quite common procedure and is not only used in DMEK but also in surgeries such as Pars‐Plana‐Vitrectomy (PPV). In the case of PPV, corneal debridement was investigated to a higher extent. It is generally considered as a safe technique. In some cases, prolonged pain and corneal scarring are reported [5]. It is also a very common technique for handling disease entities such as recurrent erosions and other ocular surface diseases [6–11].

The literature on the corneal debridement procedure in the context of DMEK is very limited. There are primarily case studies or studies with small patient cohorts. Therefore, these studies do not provide deeper insights into decisive aspects. These would primarily include visual rehabilitation as well as graft survival [12, 13]. Therefore, this study aims to provide insights into the safety and long‐term effects of this technique. By analyzing a large dataset of DMEK cases, we investigate the influence of intraoperative corneal debridement on postoperative visual acuity, endothelial cell density, graft survival, and perioperative complications. The findings of our study will contribute to the knowledge of the DMEK procedure and help guide surgical decision‐making during the process.

## 2. Methods

Approval from the university’s ethics committee was obtained beforehand (number of the ethics committee vote of the University of Freiburg: 24‐1204‐S1‐retro). Only retrospective, routine clinical data were used. All research studies were conducted in adherence to relevant ethical guidelines and regulations, ensuring compliance with institutional and international standards. In accordance with the General Data Protection Regulation (GDPR) and the German Federal Data Protection Act (BDSG), retrospective use of clinical data for research purposes is permissible without individual informed patient consent if the study serves the public interest, data protection measures such as pseudonymization or anonymization are applied, and an ethics committee has approved the research.

This retrospective comparative study analyzed 3168 eyes that underwent DMEK between 2012 and 2024 at the Eye Center of the University Hospital in Freiburg, Germany.

The study population had a mean age of 70 years (SD 10.01 years), and the mean donor age was 71 years (SD 11.2 years). Intraoperative corneal debridement was performed in 215 eyes (6.8%) based on the surgeon’s assessment of corneal clarity and the need for improved visualization during the procedure. This decision was typically made in cases of severe corneal edema, pronounced bullous keratopathy, or other epithelial irregularities that significantly obscured the surgeon’s view of the anterior chamber, thereby compromising the safe manipulation and positioning of the donor graft. While exact quantification is challenging due to variability in surgical reports, reviews of available documentation indicated that debridement was applied restrictively and consistently for these reasons, with no evidence of other relevant indications.

The primary outcome of this study was postoperative graft survival, defined as the time to graft failure requiring repeat transplantation. Secondary outcomes included the time to achieve a best‐corrected visual acuity (BCVA) of 6/12 (logMAR 0.3) or better, the time for endothelial cell density to fall below 500 cells/mm^2^, and the need for rebubbling. Survival time analyses were conducted to investigate the influence of corneal debridement on these outcomes.

Statistical analyses included both bivariate comparisons using Kaplan–Maier curves and multifactorial Cox regression to control for potential confounding factors. Kaplan–Meier survival curves were generated to assess differences in endothelial cell density, postoperative regrafting, and postoperative visual acuity. The log‐rank test was used to compare survival distributions, with the test statistic following a chi‐squared distribution (df = 1). Statistical significance was determined at a threshold of *p* < 0.005, with results reported as test statistic values and corresponding −log_2_(*p*) transformations. This approach allowed for a more comprehensive understanding of the relationship between corneal debridement and the studied outcomes. Survival analyses were performed for different indications. These were bullous keratopathy and Fuchs endothelial dystrophy.

The procedure of corneal debridement in DMEK was performed by experienced surgeons. The process of the surgery includes various steps. At first, the donor tissue is prepared, while the recipients’ Descemet membrane is stripped. Subsequently, the prepared donor tissue is implanted through small incisions in the periphery of the cornea and positioned to ensure proper alignment. The surgeon then uses an air/gas bubble to press the donor tissue against the recipient cornea, facilitating adhesion. In some cases, especially when there is *s* significant cataract, a Triple‐DMEK procedure is used where also the lens is removed and an artificial lens is implanted. The step of corneal debridement is usually performed at the beginning of the surgery and has to be decided individually. In postoperative OCT‐scans of the anterior segment of the eye as well as in slit‐lamp microscopy, it is decided whether there is significant graft detachment. In these cases, a second insertion of gas or air is the current gold standard [14–16].

## 3. Results

**Figure 1:**
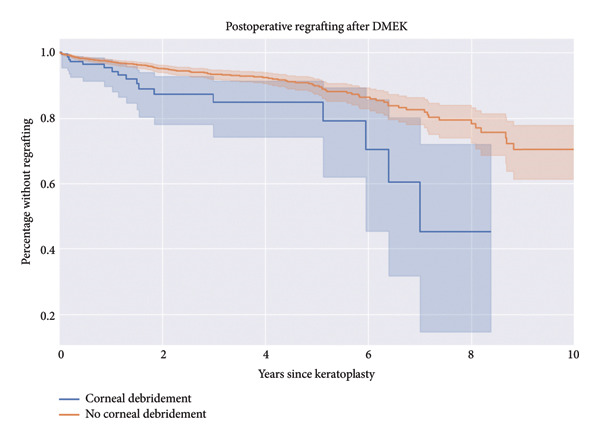
Kaplan–Meier curve for the percentage without regrafting.

In this retrospective comparative study, 3168 eyes that underwent DMEK between 2012 and 2024 were analyzed to investigate the influence of intraoperative corneal debridement on postoperative outcomes. Corneal debridement was performed in 215 eyes (6.8%) in order to improve visualization during the procedure. Table 1 shows the baseline characteristics of our study cohort. Significant differences were found between eyes that underwent corneal debridement (*N* = 215) and those that did not (*N* = 2953). Patients in the debridement group were older (median 75.0 years vs. 71.0 years, *p* < 0.001) and had fewer cases of Fuchs’ endothelial dystrophy as the surgical indication (50% vs. 90%, *p* < 0.001). Additionally, Triple‐DMEK was less frequently performed in the debridement group (35% vs. 60%, *p* < 0.001), and these patients had a shorter median follow‐up time (258 vs. 370 days, *p* = 0.015). No significant differences were observed in donor age, endothelial cell density, or days in culture medium (*p* > 0.05). Survival time analyses revealed differences in outcomes between the corneal debridement and no corneal debridement groups. The percentage of patients without requiring regrafting over 10 years was higher in the no‐debridement group compared to the debridement group​ (Figure 1). The survival analysis for postoperative regrafting revealed a significant difference, with a log‐rank test statistic of 10.73 and a *p* value of < 0.005 (−log_2_(*p*) = 9.89).

**Table 1:** Baseline statistics for our study cohort.

	*N*	Corneal debridement (*N* = 215)	Without corneal debridement (*N* = 2953)	Statistical test and metric
Age	3168	67.0/75.0/81.5	64.0/71.0/77.0	*F* = 22.78 d.f. = 1, 3166 *p* < 0.001
Female	3062	61% (120)	58% (1665)	Chi‐square = 0.59 d.f. = 1 *p* = 0.441
Indication: Fuchs	3168	50% (108)	90% (2667)	Chi‐square = 296.3 d.f. = 1 *p* < 0.001
Triple‐DMEK	3168	35% (76)	60% (1763)	Chi‐square = 48.81 d.f. = 1 *p* < 0.001
Follow‐up time	3168	29.0/258.0/673.5	50.0/370.0/841.0	*F* = 5.97 d.f. = 1, 3166 *p* = 0.015
Donor age	3168	64.0/73.0/79.0	63.0/72.0/80.0	*F* = 0.98 d.f. = 1, 3166 *p* = 0.322
Days in culture medium	3168	21.5/25.6/28.3	20.6/25.6/28.36	*F* = 0.08 d.f. = 1, 3166 *p* = 0.782
Endothelial cell density (preoperative)	3163	2190.0/2336.0/2482.0	2190.0/2336.0/2482.0	*F* = 0.12 d.f. = 1, 3161 *p* = 0.731

*Note:* Note that the difference in *N* is due to missing data.

**Figure 2:**
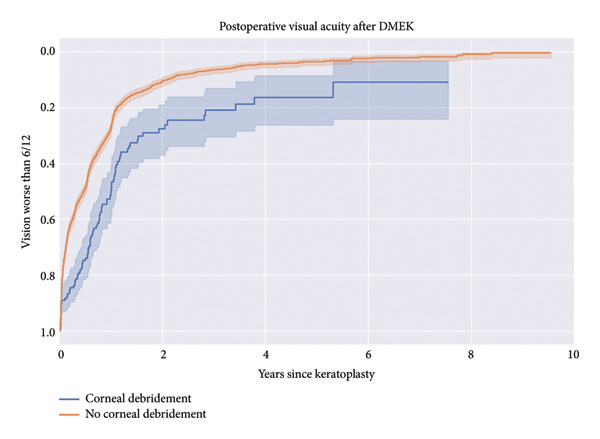
Kaplan–Meier curve for vision gain. An event was defined as achieving a best‐corrected visual acuity of 6/12 (logMAR 0.3) or better.

Similarly, the percentage of patients with vision better than 6/12 (logMAR 0.3) over 10 years was higher in the no‐debridement group (Figure 2). The Kaplan–Meier analysis for postoperative visual acuity demonstrated a highly significant difference, with a log‐rank test statistic of 35.98 and a *p* value of < 0.005 (−log_2_(*p*) = 28.9).

**Figure 3:**
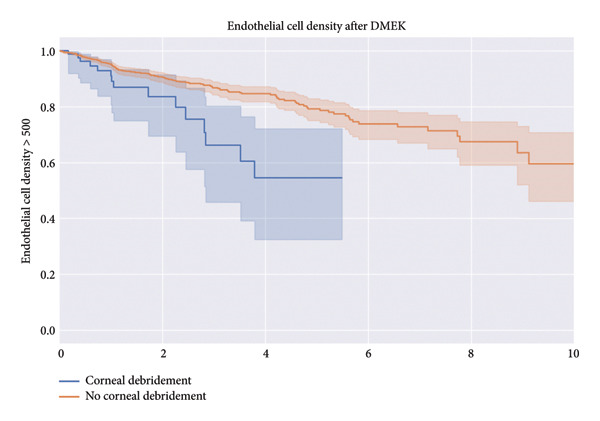
Percentage with an endothelial cell density over 500 cells/mm^2^.

The no‐debridement group also showed a higher percentage of patients without rejection episodes over 10 years (as shown in the Kaplan–Meier curve in the supporting information [available here], which is similar to the curves for other inspected topics) and a higher percentage of patients maintaining an endothelial cell density > 500 cells/mm^2^ over 10 years (Figure 3) compared to the debridement group. The log‐rank test showed a significant difference in endothelial cell density over time, with a test statistic of 9.91 and a *p* value of < 0.005 (−log_2_(*p*) = 9.25). Additionally, the percentage of patients without requiring rebubbling over 50 months was higher in the no‐debridement group (corresponding figure in supporting information [available here]). The log‐rank test comparing the need for rebubbling after DMEK between patients with and without corneal debridement showed no statistically significant difference (*χ* ^2^ = 0.44, *p* = 0.51, −log_2_(*p*) = 0.98). The results for the log‐rank test of the bivariate analyses can be found in Table 2.

**Table 2:** Statistical analysis of Kaplan–Meier curves for different metrics.

Test	Null distribution	Degrees of freedom	Test name	Test statistic	*p*	−log_2_(*p*)
Endothelial cell density	Chi‐squared	1	Log‐rank	9.91	< 0.005	9.25
Postoperative regrafting	Chi‐squared	1	Log‐rank	10.73	< 0.005	9.89
Postoperative visual acuity	Chi‐squared	1	Log‐rank	35.98	< 0.005	28.9
Need for rebubbling	Chi‐squared	1	Log‐rank	0.44	0.5	0.98

**Figure 4:**
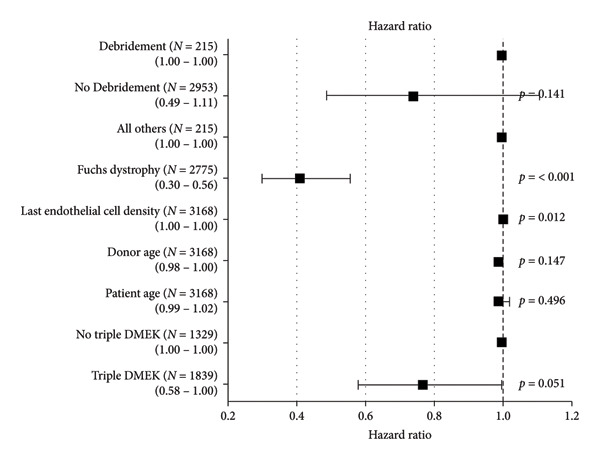
Forest plot showing the results from the multifactorial Cox‐regression.

Multifactorial Cox regression analysis was performed to control for potential confounding factors (Figure 4). The presence of corneal debridement had a hazard ratio of 0.74 (95% CI: 0.49–1.11) compared to no debridement, but the difference was not statistically significant (*p* = 0.141). Other factors, such as donor age (HR = 1.00, 95% CI: 0.99–1.02, *p* = 0.496), last endothelial cell density (HR = 0.99, 95% CI: 0.98–1.00, *p* = 0.147), and indication (Fuchs Dystrophy vs. others, HR = 0.41, 95% CI: 0.30–0.56, *p* < 0.001), were also analyzed. In summary, the bivariate survival analyses suggest that intraoperative corneal debridement during DMEK may be associated with slightly worse postoperative outcomes in terms of visual acuity, endothelial cell density, graft survival, and perioperative complications. However, when controlling for potential confounding factors in the multifactorial Cox regression analysis, the difference in hazard ratios between the debridement and no‐debridement groups was not statistically different.

Interestingly, the study found that patients with Fuchs’ endothelial dystrophy had a significantly lower risk of graft failure compared to other indications (HR 0.41, *p* < 0.001). This may be due to the generally healthier status of the host corneal stroma and the absence of prior intraocular surgeries in these patients. Additionally, patients undergoing a triple procedure (DMEK IOL (intraocular lens) explantation/implantation) also had a lower risk of graft failure (HR 0.77, *p* = 0.05), possibly due to the comprehensive management of concurrent pathologies.

## 4. Discussion

This study provides valuable insights into the safety of intraoperative corneal debridement during DMEK. While the initial bivariate analysis suggested slower visual recovery and higher retransplantation rates in the debridement group, the multifactorial Cox regression revealed that corneal debridement itself did not have statistically significant influence on graft failure risk when controlling for potential confounders. This finding highlights the importance of considering the overall clinical context when interpreting surgical outcomes.

The observed differences in the bivariate analysis may be attributed to the fact that corneal debridement is more likely to be performed in cases with advanced corneal edema or poor visualization, which are inherently more challenging and may have less favorable prognoses. The multifactorial analysis accounted for these factors, revealing that the underlying disease severity, rather than the debridement technique, likely contributed to the apparent differences in outcomes.

The lower risk of graft failure in patients with Fuchs’ endothelial dystrophy compared to other indications may be explained by the generally healthier host corneal stroma and the absence of prior intraocular surgeries in these patients. Similarly, the lower graft failure risk associated with triple procedures (DMEK + iridectomy + IOL implantation) may be due to the comprehensive management of concurrent pathologies, addressing multiple issues simultaneously.

It is important to note that this study, while providing valuable insights, has some limitations. The retrospective nature of the study and the potential for unmeasured confounding factors should be considered when interpreting the results. Future prospective studies with standardized protocols and more detailed data collection could further validate these findings and provide additional insights into the optimal management of challenging DMEK cases.

The study’s strengths, including a large sample size and a long follow‐up period, enhance the reliability of the findings. However, the retrospective nature of the study and the potential for unmeasured confounding factors should be considered when interpreting the results.

In conclusion, no explicit evidence was found raising safety concerns for intraoperative corneal debridement in DMEK, demonstrating that it does not significantly impact long‐term graft survival or visual outcomes when controlling for potential confounders. The apparent disadvantages observed in the debridement group during the bivariate analysis are possibly explained by confounding due to more severe initial conditions.

Corneal debridement, which involves mechanically removing the corneal epithelium, can be a valuable technique to improve anterior chamber visualization and facilitate graft positioning in DMEK cases with advanced corneal edema or poor visual conditions. This study provides evidence that supports the use of corneal debridement as a safe option in these challenging situations, without compromising the overall success of the procedure.

Future prospective studies with standardized protocols and more detailed data collection could further validate these findings and provide additional insights into the optimal management of challenging DMEK cases. Such studies may also help identify specific patient subgroups that are most likely to benefit from corneal debridement and guide surgical decision‐making.

In summary, this study contributes to the growing body of knowledge on DMEK and supports the safety of intraoperative corneal debridement in selected cases. Surgeons can consider this technique as a viable option to improve visualization and facilitate graft positioning compromising the long‐term outcomes of the procedure. However, careful patient selection and individualized risk assessment remain essential to optimize outcomes in DMEK.

## Disclosure

The manuscript is based on the preprint by Arens et al. [17].

## Conflicts of Interest

The authors declare no conflicts of interest.

## Author Contributions

S.A. conceived the study, led data acquisition and interpretation, and drafted all manuscript sections. S.J.L. provided statistical expertise, guided survival analysis and Cox regression, and ensured methodological rigor. He also reviewed the manuscript. D.B. architected the dataset, overseeing data extraction, cleaning, and organization. He provided expertise in clinical application and manuscript review. T.R. provided the initial idea for the study, senior oversight, clinical expertise, and critical review of the study design and interpretation. He also participated in manuscript review.

## Funding

Open Access funding enabled and organized by Projekt DEAL.

## Supporting Information

Supporting Figure 1: Percentage of patients who required rebubbling after DMEK, with a higher percentage of patients in the no‐debridement group avoiding the need for rebubbling over 50 months. The log‐rank test showed no statistically significant difference between the groups (*p* = 0.51).

## Supporting information


**Supporting Information** Additional supporting information can be found online in the Supporting Information section.

## Data Availability

The raw data used in this research may contain identifying information. However, upon request, I am happy to provide access to the data while ensuring that appropriate measures are taken to protect privacy and confidentiality. Please contact the corresponding author Sebastian Arens (sebastian.arens@uniklinik-freiburg.de) in case data access is required.
