# Supplementary figures and images for: Safety Profile of Intraoperative Corneal Debridement in Descemet Membrane Endothelial Keratoplasty (DMEK)—A Retrospective Comparative Study
Source: J Ophthalmol. 2025 Dec 19;2025:6694690. doi: 10.1155/joph/6694690 (PMC12767040; doi:10.1155/joph/6694690)

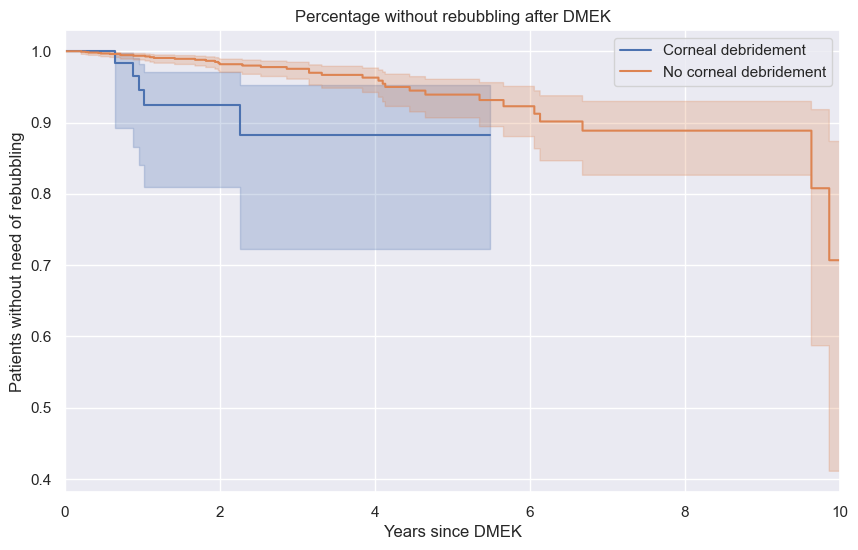

Supplement: Supplementary file 1 — Supporting Information Additional supporting information can be found online in the Supporting Information section. [file JOPH-2025-6694690-s001.zip › rebubbling.png]

Percentage without rebubbling after DMEK

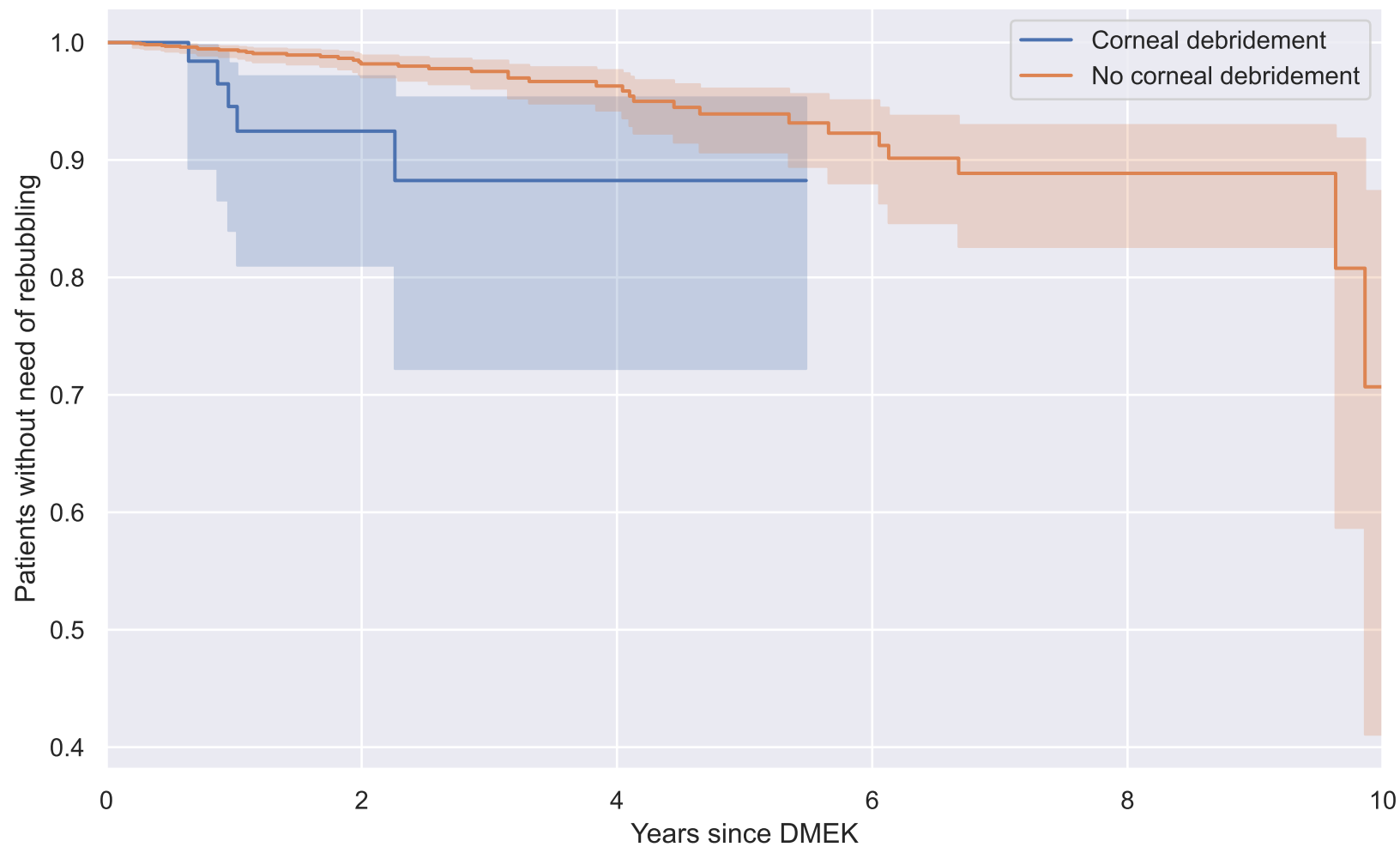

Supplement: Supplementary file 1 — Supporting Information Additional supporting information can be found online in the Supporting Information section. [file JOPH-2025-6694690-s001.zip › rebubbling_editable.pdf]
